# On the automatic link between affect and tendencies to approach and avoid: Chen and Bargh (1999) revisited

**DOI:** 10.3389/fpsyg.2015.00335

**Published:** 2015-04-02

**Authors:** Mark Rotteveel, Alexander Gierholz, Gijs Koch, Cherelle van Aalst, Yair Pinto, Dora Matzke, Helen Steingroever, Josine Verhagen, Titia F. Beek, Ravi Selker, Adam Sasiadek, Eric-Jan Wagenmakers

**Affiliations:** ^1^Social Psychology Program, Department of Psychology, Faculty of Behavioral and Social Sciences, University of AmsterdamAmsterdam, Netherlands; ^2^Amsterdam Brain and Cognition, University of AmsterdamAmsterdam, Netherlands; ^3^Cognitive Neuroscience GroupAmsterdam, Netherlands; ^4^Psychological MethodsAmsterdam, Netherlands

**Keywords:** emotion, approach, avoidance, replication, affect

## Abstract

Within the literature on emotion and behavioral action, studies on approach-avoidance take up a prominent place. Several experimental paradigms feature successful conceptual replications but many original studies have not yet been replicated directly. We present such a direct replication attempt of two seminal experiments originally conducted by Chen and Bargh (1999). In their first experiment, participants affectively evaluated attitude objects by pulling or pushing a lever. Participants who had to pull the lever with positively valenced attitude objects and push the lever with negatively valenced attitude objects (i.e., congruent instruction) did so faster than participants who had to follow the reverse (i.e., incongruent) instruction. In Chen and Bargh's second experiment, the explicit evaluative instructions were absent and participants merely responded to the attitude objects by either always pushing or always pulling the lever. Similar results were obtained as in Experiment 1. Based on these findings, Chen and Bargh concluded that (1) attitude objects are evaluated automatically; and (2) attitude objects automatically trigger a behavioral tendency to approach or avoid. We attempted to replicate both experiments and failed to find the effects reported by Chen and Bargh as indicated by our pre-registered Bayesian data analyses; nevertheless, the evidence in favor of the null hypotheses was only anecdotal, and definitive conclusions await further study.

## Introduction

Several prominent psychological theories (Frijda, 1986, 2007; Lang and Bradley, 2008) state that at the core of an emotion is the tendency to act. In order to survive, organisms need to approach reward and avoid danger or punishment. These tendencies to act, it is argued, manifest themselves in part through emotions. Other psychological theories also assume a link between action and evaluation (e.g., Lang et al., 1990; Cacioppo and Gardner, 1999; Neumann et al., 2003; Strack and Deutsch, 2004). Empirically, there is ample evidence for an association between affective evaluation and some kind of approach and avoidance behavior (e.g., Solarz, 1960; Chen and Bargh, 1999; Rotteveel and Phaf, 2004; Krieglmeyer and Deutsch, 2010). What remains unclear, however, is the precise nature of this association; some researchers have argued that it is basic, direct, and default (e.g., Chen and Bargh, 1999; Duckworth et al., 2002), whereas others have argued that the association is more flexible and goal orientated (e.g., Bamford and Ward, 2008). This debate was initiated with the publication of the article by Chen and Bargh (1999) entitled “Consequences of automatic evaluation: Immediate behavioral predispositions to approach or avoid the stimulus.”

In the field of emotion and approach-avoidance behavior, the Chen and Bargh (1999) article (henceforth CB) has attracted a lot of attention (cited 866 times, January 19th 2015). In their first experiment, one that may be considered a conceptual replication of an earlier experiment (Solarz, 1960, cited 214 times, January 19th 2015), CB instructed participants to evaluate attitude objects affectively by pulling or pushing a lever. Participants who had to pull the lever for positively valenced attitude objects and push the lever for negatively valenced attitude objects did so faster than participants who had to pull for negatively valenced attitude objects and push for positively valenced attitude objects. In other words, the results showed a congruency effect between the affective valence of attitude objects and the direction of the lever movement—it is easier to pull (i.e., execute a “toward yourself” movement) for positively valenced objects and to push (i.e., execute an “away-from yourself” movement) for negatively valenced objects.

In their second experiment, CB manipulated congruency within participants and eliminated the explicit evaluative instruction. Specifically, participants were instructed to respond to the mere presentation of the attitude objects; in one block of trials, participants had to push the lever (i.e., execute an “away-from yourself” movement), and in another block they had to pull the lever (i.e., execute a “toward yourself” movement). The results again demonstrated a congruency effect: pulling was faster for positively valenced attitude objects, and pushing was faster for negatively valenced attitude objects. On basis of these results, CB concluded that (1) attitude objects are automatically evaluated; and (2) attitude objects automatically trigger a behavioral tendency to approach or avoid.

Since the publication of CB, numerous papers have been published in which approach and avoidance behavior was studied; however, the automatic link between affective evaluation and approach-avoidance tendencies was often simply taken for granted. To complicate matters, different results have been obtained using different experimental paradigms such as the manikin task (e.g., De Houwer et al., 2001), the joystick task (e.g., Eder and Rothermund, 2008), the joystick with zoom task (e.g., Rinck and Becker, 2007), and a button stand task (e.g., Rotteveel and Phaf, 2004). One issue with such conceptual replications of approach and avoidance behavior concerns construct validity. That is, different operationalizations in conceptual replications can tap different psychological processes reflecting partially different constructs that vary in their relation with the conceptualization of approach and avoidance as in the CB studies.

Replication is at the core of the scientific effort to further our understanding of the empirical world. Many effects do replicate reliably across laboratories in psychology (e.g., Simons, 2014), but some prominent effects are now under doubt (e.g., Pashler and Wagenmakers, 2012). Although opinions differ with regard to the extent of this “replication crisis” (e.g., Pashler and Harris, 2012; Stroebe and Strack, 2014), the scientific community seems to be shifting its focus more toward direct replication. For instance, several journals recently proposed special issues on replication (e.g., Nosek and Lakens, 2014, this issue of *Frontiers in Psychology*) or even launched a new type of article (i.e., Registered Replication Reports in *Cortex, Perspectives on Psychological Science, Attention, Perception, and Psychophysics*, and other journals (see Chambers, 2013; Wolfe, 2013); for an overview see https://osf.io/8mpji/wiki/home/).

Direct replications benefit from preregistration of design and analysis plan, ensuring a clean separation between which analyses are pre-planned (i.e., confirmatory, hypothesis-testing) and which analyses are *post-hoc* (i.e., exploratory, hypothesis-generating; see e.g., De Groot, 1956/2014; Wagenmakers et al., 2012). Such separation is also required for the proper statistical interpretation of the results. When an initial finding replicates successfully in a preregistered setting, this raises researchers' confidence that the effect is real and can form the basis for more empirical as well as theoretical work. When an initial finding fails to replicate, however, scientific effort may be re-oriented toward other, more promising avenues of investigation—at least when null results are published and do not disappear in the file drawer (e.g., Rosenthal, 1979; Francis, 2013). Direct replications not only affect one's confidence about the veracity of the phenomenon under study, but they also increase our knowledge about effect size (see also Simons, 2014; but see also Stroebe and Strack, 2014).

**Figure 1:**
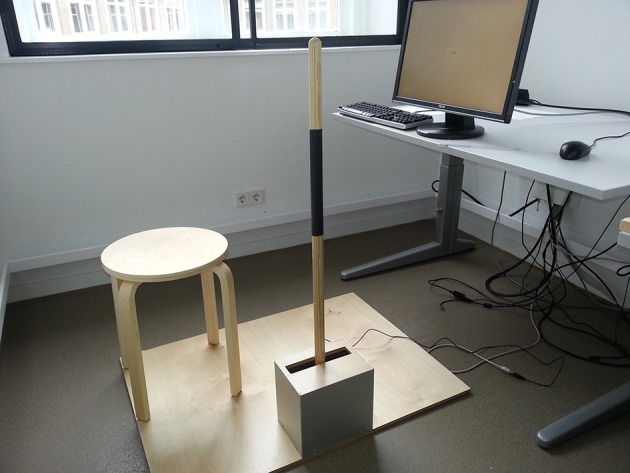
**The experimental setup of Experiment 1 and Experiment 2**. The 100 cm lever is fixed to base with a hinge. Two identical weak springs make sure that the lever will return to mid position after responding. Responses were recorded whenever the lever reached 15.6° of movement backwards and 15.3° of movement forwards.

Our decision to replicate the CB studies was motivated in part by a recent meta-analysis on approach and avoidance behavior including 29 published studies and 81 effect sizes (Phaf et al., 2014), which indicated a moderate publication bias for congruency effects with explicit affective evaluation as obtained in Experiment 1 of CB. More importantly, to the best of our knowledge the CB findings were never replicated directly. This is remarkable, particularly in light of the central importance of the CB findings in the literature on emotion and approach and avoidance behavior. For these reasons we attempted to replicate the original CB findings using a similar experimental setup (i.e., a lever, see Figure 1), similar stimuli, and similar instructions. To remove all researcher's degrees of freedom in the analysis stage we used a preregistered protocol on the Open Science Framework^1^ (e.g., Open Science Collaboration, 2012). This protocol detailed the design, method, hypotheses, as well as the entire analysis plan.

In direct replication studies it is essential to be able to quantify evidence in favor of the null hypothesis. In addition, it is desirable to collect data until the results are compelling. Neither desideratum can be accomplished within the framework of frequentist statistics, and this is why our analysis of both experiments will rely on hypothesis testing using the Bayes factor (e.g., Edwards et al., 1963; Berger and Mortera, 1999; Wagenmakers, 2007; Rouder et al., 2009, 2012; Wagenmakers et al., 2012). The method section below provides the details of our design and analysis methodology. This research follows a strictly confirmatory protocol as described in Wagenmakers et al. (2012).

## Experiment 1

### Method

#### Pre-registered sampling plan

**Figure 2:**
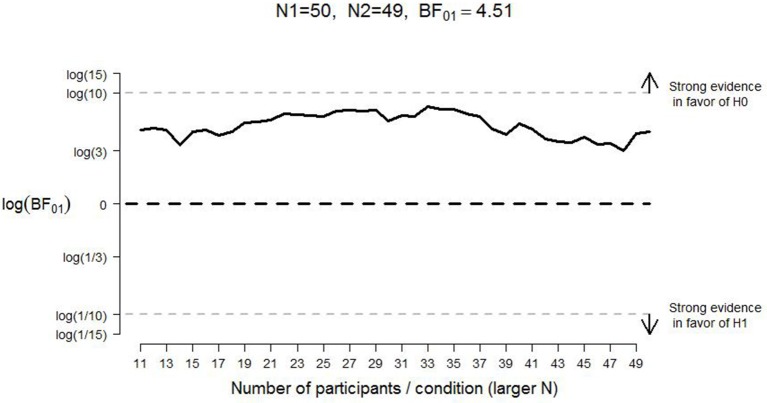
**Development of the log Bayes factor as a function of the number of participants per condition for good judgments in Experiment 1**. N1 = sample size in the positive-pull condition; N2 = sample size in the positive-push condition; *BF*_01_ = Bayes factor in favor of the null hypothesis; H0 = null hypothesis; H1 = alternative hypothesis. The horizontal black dashed line indicates complete ambiguous evidence, and the horizontal dashed gray lines indicate strong evidence either in favor of the null hypothesis [i.e., log(*BF*_01_) ≥ log(10)] or in favor of the alternative hypothesis [i.e., log(*BF*_01_) ≤ log(1/10)].

**Figure 3:**
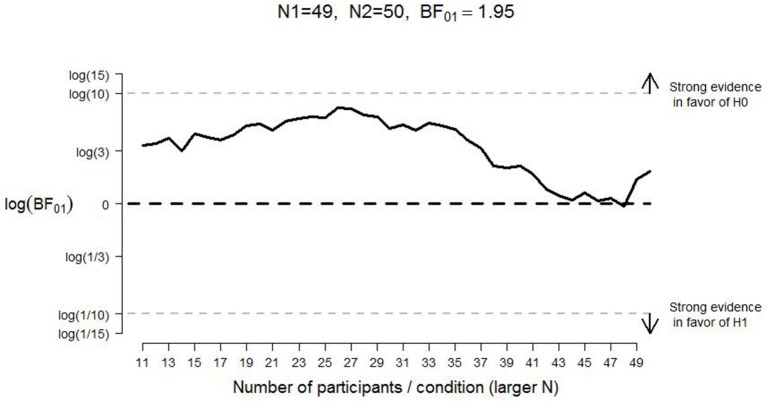
**Development of the log Bayes factor as a function of the number of participants per condition for bad judgments in Experiment 1**. N1 = sample size in the negative-pull condition; N2 = sample size in the negative-push condition; *BF*_01_ = Bayes factor in favor of the null hypothesis; H0 = null hypothesis; H1 = alternative hypothesis. The horizontal black dashed line indicates complete ambiguous evidence, and the horizontal dashed gray lines indicate strong evidence either in favor of the null hypothesis [i.e., log(*BF*_01_) ≥ log(10)] or in favor of the alternative hypothesis [i.e., log(*BF*_01_) ≤ log(1/10)].

A frequentist analysis would start with an assessment of the effect size of Experiment 1 from CB which would then form the basis of a power analysis to determine the number of participants that yields a specific probability for rejecting the null hypothesis when it is false. This frequentist analysis plan is needlessly constraining and potentially wasteful: the experiment cannot continue after the planned number of participants has been tested, and it cannot stop even when the data yield a compelling result earlier than expected (e.g., Wagenmakers, 2007). Here we circumvent these frequentist limitations by calculating and monitoring the Bayes factor (e.g., Edwards et al., 1963; Berger and Mortera, 1999; Wagenmakers et al., 2012; Rouder et al., 2012). The Bayes factor quantifies the change from prior model odds to posterior model odds; in other words, the Bayes factor quantifies the extent to which the data shift our opinion away from one hypothesis and toward another. A Bayes factor of 5 in favor of the null hypothesis, for example, indicates that the data are 5 times more likely to occur under the null hypothesis than under the alternative hypothesis. For the interpretation of evidence in the Bayesian paradigm, the intention with which the data are collected is irrelevant; hence, the Bayes factor can be monitored as the data come in, and data collection can be terminated at any point (Berger and Wolpert, 1988; Rouder, 2014; see also Figures 2, 3).

Based on the above considerations, our sampling plan was as follows: We set out to collect a minimum of 20 participants in each between-subject condition (i.e., the congruent and incongruent condition, for a minimum of 40 participants in total). Next we planned to monitor the Bayes factor and stop the experiment whenever both critical hypothesis tests (detailed below) reached a Bayes factor that could be considered “strong” evidence (Jeffreys, 1961); this meant that the Bayes factor should be either 10 in favor of the null hypothesis, or 10 in favor of the alternative hypothesis. The experiment was also to be stopped whenever the maximum number of participants was reached. This number was set to 50 participants per condition (i.e., a maximum of 100 participants in total). Additionally, the experiment was to be stopped by January 1st, 2014 if neither criteria were met. The latter date was, however, amended on OSF on January the 7th and reset to January 31st 2014 because the Bayes factor had not reached the pre-set level of strong evidence and the maximum number of participants had not been reached either. From a Bayesian perspective the specification of this sampling plan was needlessly precise; we nevertheless felt the urge to be as complete as possible. In the end, data collection was terminated because the maximum number of participants was reached.

#### Participants

We recruited 100 students (23 male, mean age = 21.2 year, SD = 0.42; Congruent: 10 male; Incongruent: 13 male) from the University of Amsterdam. All participants were rewarded with course credits or €5. Only students with Dutch as their native language were allowed to participate. One participant did not meet this criterion and was excluded from further analysis. All participants were informed about the procedure with an information brochure and subsequently signed an informed consent form.

#### Materials

Participants were seated in a dimly lit room at approximately one arm length's distance in front of a computer monitor. A 100 cm long lever (see Figure 1) triggered one of two switches upon being pulled or pushed for, respectively15.6 or 15.3° (Note that CB report a movement of 10° in both directions with a lever measuring 92 cm). Participants held the lever at a marked height of approximately 68 cm resulting in actual hand movement of 18.7 cm forwards and 19 cm backwards. In CB's experiments actual hand movements were 12 cm in both directions assuming participants were holding the lever at the same height as in our experiment^2^. Both switches were connected to the computer so that response latencies as well as movement direction could be recorded. Responses were recorded using a mechanism based on a Logitech G400 gaming mouse. Polling rate of the mouse was set at 500 S/s, response latency was less than 3 ms. Start of lever movement was recorded by polling the mouse position every screen refresh (16.667 ms). This means start-of-response latency could be anywhere between 3 and 16.67 ms. To ensure that the lever returned to the central position after each response, two identical weak springs were connected to the lever at the front and at the back. These springs were added to the experimental setup to make sure that the lever would return to mid position after responding. On the few occasions that the lever was not returning to mid position by itself, participants were instructed to move the lever back to mid position themselves. We used almost the same attitude objects (henceforth “targets”) as were used in the original CB study and reported in Bargh et al. (1992). The targets were first translated (see Tables 1, 2 in Supplementary Material) from English to Dutch, and then back-translated to check for any inconsistencies. Inconsistent targets were replaced with comparable alternates in order to approach the original stimuli as closely as possible in Dutch (i.e., “hangover” was replaced with “misselijkheid” which means “nausea”; “Reagan” was replaced with the name of the current Dutch prime-minster “Rutte”). Students from the University of Amsterdam (*n* = 130) then evaluated these targets in a test session on an 11 point scale (from very bad −5 to very good +5) and the resulting mean evaluations were compared to the mean evaluations of the original stimuli (Bargh et al., 1992). Targets that differed substantially in their affective evaluation (e.g., “beer”; “priest”; “clown”) were excluded, as well as targets that differed more than 1.5 points on average from the original ratings of Bargh et al. (1992; see Table 2 in Supplementary Material). As can be expected, the valence of the remaining 78 targets (i.e., 39 positive targets and 39 negative targets) correlated highly with the affective valence of the original stimulus list (*r* = 0.98). Due to a programming mistake, the negative target “wormen” (“worms”) was replaced by the positive target “stereo” (“stereo”), a word from the practice session. Data obtained with “stereo” was excluded from further analysis so in the end responses to 39 positive words and 38 negative words were included in the results. These targets were presented in a random order and every target was presented only once in a lower case “Times” font on a white background.

#### Procedure

After reading the information brochure and signing an informed consent form, participants were seated in front of the computer screen with the lever next to their dominant hand, after which they read the procedure of the experiment off the screen. Participants were asked to classify the targets presented on the screen as either “good” or “bad” (for the exact wording of the specific lever movement instructions see Appendix A in Supplementary Material). Participants had to do this by either pushing the lever away from themselves, or pulling the lever toward themselves. The participants were alternately assigned to either the congruent or the incongruent condition. In the congruent condition, participants were instructed to pull the lever toward themselves if the target had a “good” meaning and push it away from themselves to indicate a “bad” meaning. In the incongruent condition, these instructions were reversed and participants had to pull the lever toward themselves if the target had a “bad” meaning and push it away from themselves to indicate a “good” meaning. Participants were instructed to release the lever after responding in order for it to return to its starting position. In case the lever did not return to its starting mid position itself participants were asked to return the lever themselves to its starting position.

Before the start of the actual experiment we confirmed that participants understood the instructions correctly by making them perform 10 practice trials with 10 separate targets that were not part of the 78 target words (except for “stereo,” see Table 2 in Supplementary Material). After the practice trials the experimenter left the room, so that the actual trials were completed without the experimenter present.

During the experiment each target appeared on screen until the participant pulled or pushed the lever above the 15.6 and 15.3° angle, respectively, necessary to trigger the response-switches. The computer recorded the time delay between the appearance of the target, the onset of the lever movement, and the triggering of the response-switch as specified before. The computer also recorded whether the lever had been pulled or pushed. After every response the target disappeared and it took 4 s until the next trial commenced and a new target appeared again at the center of the screen. The targets were presented in a random order with every target appearing once only. After responding on the last trial the experimenter returned to the room to thank and debrief the participant.

#### Preregistered data analysis and presentation of results

Based on the reasoning of CB and our own pilot tests, all trials with latencies greater than 3000 ms or smaller than 300 ms were excluded from further analysis (pulling with “good” judgments: 1.7%; pulling with “bad” judgments: 2.2%; pushing with “good” judgments: 1%; pushing with “bad” judgments: 1.2%). These criteria for outlier removal had been specified in the preregistration document. Whereas CB removed only latencies greater than 4000 ms, we had to reduce this value because pilot testing showed that in our setup one can easily push/pull the lever under 4000 ms. As in CB, and as specified in the preregistration document, the dependent measure for all analyses was the mean log(10)-transformed response latency for every participant. Results are reported as untransformed response latencies. The crucial hypothesis concerns the interaction that describes the congruency effect. Specifically, the congruency effect can be decomposed in two directional hypotheses: the first hypothesis states that participants respond faster to a positive target by pulling instead of pushing a lever; the second hypothesis states that participants respond faster to a negative target by pushing instead of pulling a lever. As specified in the preregistration document, the two crucial hypotheses will be assessed separately by means of two default Bayes factors for unpaired, one-sided *t*-tests as outlined in Rouder et al. (2009) and Wetzels et al. (2009). Specifically, the effect sizes under the alternative hypothesis are assumed to follow a folded Cauchy(0,1) distribution. Exploratory analyses will vary the shape of this prior to probe the robustness of our conclusions.

As described above, Bayes factors quantify the support that the data provide for the null hypothesis vis-a-vis the alternative hypothesis. Support in favor of the alternative hypotheses constitutes support in favor of the effects reported by CB in their Experiment 1.

### Results

#### Bayes factor hypothesis tests

For “good” evaluations, pulling the lever was a little faster (*M* = 1147 ms, *SE* = 29) than pushing (*M* = 1165 ms, *SE* = 30, see Table 1) whereas for “bad” evaluation, pushing the lever was (*M* = 1204 ms, *SE* = 35) faster than pulling (*M* = 1267, *SE* = 39). The direction of these effects is consistent with the results reported by CB. However, the Bayes factor (assuming equal variances, as was done for all analyses reported in this manuscript) indicated that the observed data were more likely under the null hypothesis than under the alternative hypothesis; specifically, *BF*_*01*_ = 4.51 for “good” evaluations (i.e., the data for “good” evaluations are almost five times more likely under the null hypothesis than under the alternative hypothesis) and *BF*_*01*_ = 1.95 for “bad” evaluations (i.e., the data for “bad” evaluations are almost twice as likely under the null hypothesis than under the alternative hypothesis). Figures 2, 3 display the development of the log Bayes factor as a function of the number of participants per condition for the “good” and the “bad” evaluations, respectively. Log Bayes factors larger than zero provide evidence for the null hypothesis; log Bayes factors smaller than zero provide evidence for the alternative hypothesis. For “good” evaluations, after testing 11 participants per condition, the Bayes factor fluctuated around three, indicating anecdotal to moderate evidence in favor of the null hypothesis (see Jeffreys, 1961, for a categorization of the evidential strength provided by the Bayes factor). For “bad” evaluations, the Bayes factor initially indicated moderate evidence in favor of the null hypothesis. However, after testing 27 participants per condition, the Bayes factor gradually decreased, and indicated only anecdotal evidence in favor of the null hypothesis at the end of the data collection.

**Table 1:** Response latencies in ms (SE) for lever movement in Experiment 1 and for Experiment 2 (^*^ in Experiment 1 response latencies reflect good vs. bad judgments whereas response latencies in Experiment 2 reflect responses to good vs. bad words).

	**Experiment 1**		**Experiment 2**	
	**Pull**	**Push**	**Pull**	**Push**
Good^*^	1147 (29)	1165 (30)	562 (13)	571 (14)
Bad^*^	1267 (39)	1204 (35)	574 (12)	562 (13)

#### Exploratory analysis

**Figure 4:**
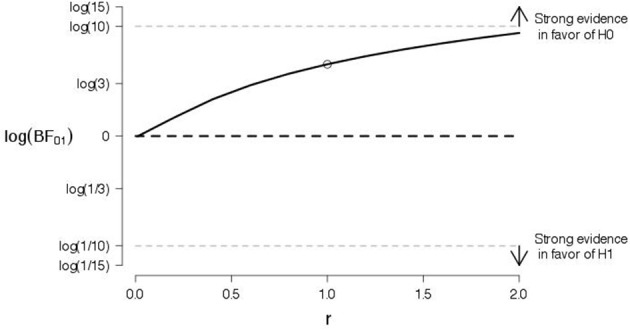
**A robustness analysis for the data of good judgments from Experiment 1**. The log Bayes factor log(*BF*_01_) is plotted as a function of the scale parameter r of the Cauchy prior for the effect size under the alternative hypothesis. The dot indicates the result from the default prior, the horizontal black dashed line indicates complete ambiguous evidence, and the horizontal dashed gray lines indicate strong evidence either in favor of the null hypothesis [i.e., log(*BF*_01_) ≥ log(10)] or in favor of the alternative hypothesis [i.e., log(*BF*_01_) ≤ log(1/10)].

**Figure 5:**
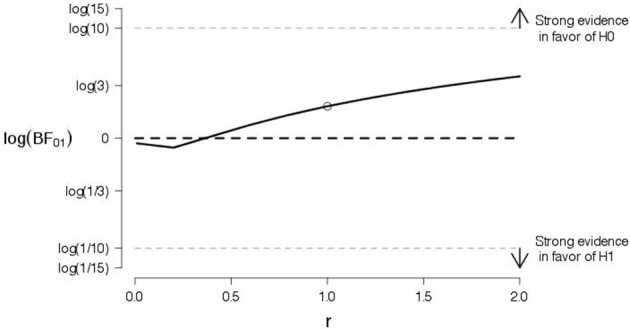
**A robustness analysis for the data of bad judgments from Experiment 1**. The log Bayes factor log(*BF*_01_) is plotted as a function of the scale parameter r of the Cauchy prior for the effect size under the alternative hypothesis. The dot indicates the result from the default prior, the horizontal black dashed line indicates complete ambiguous evidence, and the horizontal dashed gray lines indicate strong evidence either in favor of the null hypothesis [i.e., log(*BF*_01_) ≥ log(10)] or in favor of the alternative hypothesis [i.e., log(*BF*_01_) ≤ log(1/10)].

To probe the robustness of our conclusions, we varied the shape of the prior for the effect size under the alternative hypothesis. Figures 4, 5 show the log Bayes factor as a function of the scale parameter r of the Cauchy prior for the “good” and “bad” evaluations, respectively. The dot indicates the result from the default prior used in the preregistered data analysis. It is evident that, as the scale parameter r increases (i.e., the prior becomes progressively wider), the Bayes factor increasingly favors the null hypothesis. In addition, it is evident that, even under the prior setting that favors the alternative hypothesis most (i.e., scale parameter r very close to zero), the log Bayes factor is close to zero indicating ambiguous evidence.

For completeness, we also analyzed the data using a frequentist repeated measures 2 (Evaluation: “Good” vs. “Bad”) × 2 (Instruction: Congruent vs. Incongruent) analysis of variance (ANOVA). Although congruent lever movements were faster (*M* = 1176 ms, *SE* = 31) than incongruent lever movements (*M* = 1216 ms, *SE* = 33), this difference did not reach significance [*F*_(1, 97)_ < 1, *n.s*.]. Additionally, “good” evaluations were given faster than “bad” evaluations [*F*_(1, 97)_ = 36.52, *p* < 0.005, η^2^_*p*_ = 0.274; *M*_*good*_ = 1156 ms, *SE*_*good*_ = 21; *M*_*bad*_ = 1236 ms, *SE*_*bad*_ = 26]. This main effect of judgment was the opposite of that obtained by CB (i.e., “bad” evaluations were faster than “good” evaluations). As shown in Table 1, this main effect of evaluative judgment was qualified by a two-way interaction between evaluative judgment and lever movement that almost reached the 0.05 level of significance [*F*_(1, 97)_ = 3.02, *p* = 0.085; η^2^_*p*_ = 0.030]: Pulling the lever with “good” evaluations was somewhat faster than pushing [*F*_(1, 97)_ = 0.18, *n.s*.], whereas pulling the lever with negative words was somewhat slower than pushing [*F*_(1, 97)_ = 1.38, *p* = 0.243], perhaps providing a weak indication of congruency (i.e., the alternative hypothesis) as obtained in CB. No other effects reached the 0.10 level of marginal significance.

The importance of the Two-Way interaction was also assessed with the help of a Bayesian ANOVA (Rouder et al., 2012) with participants as a random factor, which equals a repeated measures ANOVA. The Bayes factor of interest contrasts the full model that includes both the main effects and the interaction to a simpler model that includes only the main effects. The Bayes factor indicates that the data support the two models to an equal extent (i.e., *BF*_*01*_ = 1.20).

In sum, our preregistered Bayesian hypothesis tests yielded evidence in favor of the null hypothesis, although the strength of this evidence was not compelling. The exploratory Bayesian ANOVA suggested that the data do not favor the alternative hypothesis over the null hypothesis. From a Bayesian perspective, the data certainly did not support the hypothesis as proposed by CB although our experiment included almost twice as many participants (*n* = 52 in Experiment 1 of CB). Our *post-hoc* frequentist data analysis, however, did indicate some weak evidence in favor of the alternative hypothesis, consistent with the original CB findings. Although we did not find a general congruency effect as reported by CB we did obtain an interaction indicative of a similar congruency between affective evaluation and lever movement.

The discrepancy between the outcome of the frequentist and Bayesian hypothesis tests arguably reflects the shortcomings of *p*-value based null hypothesis significance testing. Despite its widespread use, most psychologists fail to recognize that *p*-values overestimate the amount of statistical evidence against the null hypothesis (e.g., Berger and Delampady, 1987; Wagenmakers, 2007; Wetzels et al., 2011; Johnson, 2013). When researchers compute *p*-values, they only consider the plausibility of the data given the null hypothesis and ignore the possibility that the data may be similarly implausible given the alternative hypothesis (Berkson, 1938; Wagenmakers et al., 2015). Note also that, contrary to Bayes factors, *p*-values cannot be used to quantify evidence in favor of the null hypothesis; within the frequentist framework one can only fail to reject the null. The fact that Bayes factors can be used to obtain evidence for the absence of a hypothesized effect makes Bayes inference particularly useful for assessing the success of replication studies.

**Figure 6:**
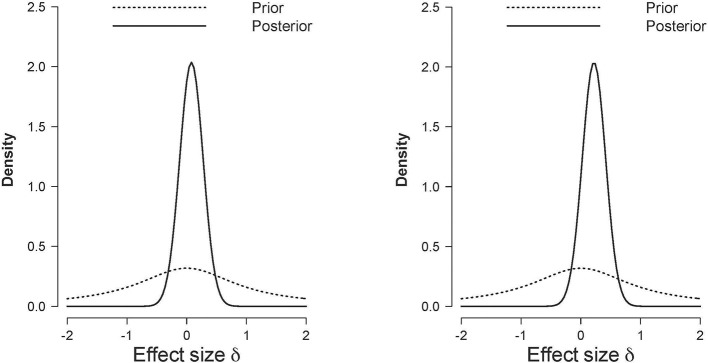
**Prior (dashed line) and posterior (solid line) distribution of the effect size d in Experiment 1 for positive judgments (left panel) and negative judgments (right panel)**. The horizontal black dashed line indicates complete ambiguous evidence, and the horizontal dashed gray lines indicate strong evidence either in favor of the null hypothesis [i.e., log(*BF*_01_) ≥ log(10)] or in favor of the alternative hypothesis [i.e., log(*BF*_01_) ≤ log(1/10)].

In comparison with the (corrected for publication bias) small to medium sized effect size reported in Phaf et al. (2014) the effect size in this experiment seems very low even though we tested almost twice as many participants than CB did. Figure 6 shows the posterior distribution of the effect size for the two pre-registered comparisons (i.e., for “good” evaluations in the left panel, and for “bad” evaluations in the right panel). The posterior distribution quantifies the uncertainty about the effect size given the observed data. It is evident, that in the case of the “good” evaluations, most posterior mass is around 0; in the case of the “bad” evaluations, the posterior distribution is slightly shifted to positive values.

## Experiment 2

### Method

#### Pre-registered sampling plan

A frequentist analysis would start with an assessment of the effect size of Experiment 2 from CB which would then form the basis of a power analysis. As for Experiment 1, however, our analysis is based on monitoring the Bayes factors of the critical hypothesis tests (detailed below).

Specifically, our sampling plan was as follows: We first set out to collect a minimum of 30 participants in a within-subject design. Next we planned to monitor the Bayes factors and stop the experiment whenever both critical hypothesis tests (detailed below) reached a Bayes factor that could be considered “strong” evidence (Jeffreys, 1961); this means that the Bayes factor should be either 10 in favor of the null hypothesis, or 10 in favor of the alternative hypothesis. The experiment would also stop whenever we would first reach the maximum number of participants, which we set to 50 participants. Finally, the experiment would also stop on January 1st, 2014, in case neither of the two other criteria had been met. As was the case for Experiment 1, data collection for Experiment 2 was terminated because the maximum number of participants was reached.

#### Participants

We recruited 56 students from the University of Amsterdam. Six participants were excluded for the following reasons: three participants did not operate the lever as instructed; two participants did not receive the correct instructions due to technical failure; and one left-handed participant was excluded because the experimental setup was not positioned correctly (i.e., at the left side). The remaining 50 participants (10 male, mean age = 21.3 year, SD = 3.5) were all native Dutch speakers and had not participated in Experiment 1. Participants were rewarded with course credits or €5. All participants were informed about the procedure with an information brochure and subsequently signed an informed consent form.

#### Materials and procedure

The same materials used in Experiment 1 were also used in Experiment 2, except that “worms” was now included in the stimulus set, resulting in a total of 78 targets (i.e., 39 positive targets and 39 negative targets). The procedure differed only with respect to instructions given (See Appendix B in Supplementary Material, instructions a and b). In contrast to Experiment 1, participants were not instructed to evaluate the targets affectively. Instead, and in accordance with CB, participants were told that the experiment was about responding as quickly as possible to the mere presentation of the words. We alternately assigned participants to a condition in which they either always pushed the lever away from themselves (Instruction a) or always pulled the lever toward themselves (Instruction b). To discourage anticipation based on timing, targets were presented after a random delay from 2 to 7 s. The original study by CB did not include such random delays. We added this feature to the design in order to prevent timed responding based on fixed delays.

After half of the trials had been completed, a text appeared on screen to inform the participants that instructions would now change and that they had to switch lever movement direction from pushing to pulling (or vice versa). Additionally, the experimenter returned to the room to explain the new instructions and to ensure that the participants had understood them. Across all participants in CB's Experiment 2, the targets were presented in a fixed order. Although not explicated in CB, this may have been done to ensure the presence of an equal number of positive and negative objects as well as an equal number of weak attitude objects in both conditions (for details see CB). We followed these constraints but presented our targets in a semi-random fashion; targets were randomly drawn without replacement from two different lists containing 19 positive targets and 20 negative targets or 20 positive targets and 19 negative targets, respectively. For every participant the order of both lists was the same.

#### Preregistered data analysis and presentation of results

Our data analysis closely followed that of Experiment 1, the main exception being that the design was fully within-subjects instead of between-subjects with regard to the association between affective valence of the targets and specific lever movement. As outlined in the preregistration document, we followed the reasoning of CB and treated response times above 1500 ms and below 300 ms as outliers, and excluded them from the analysis (pulling with positive words: 2.4%; pulling with negative words: 1.2%; pushing with positive words: 1.6%; pushing with negative words: 1.7%). As in Experiment 1, and as outlined in the preregistration document, the dependent measure was the mean log(10)-transformed response latency for every participant. The crucial hypothesis (i.e., alternative hypothesis) concerned the interaction that describes the congruency effect. Specifically, the congruency effect can be decomposed in two directional hypotheses: the first hypothesis states that participants respond faster to a positive target by pulling instead of pushing a lever; the second hypothesis states that participants respond faster to a negative target by pushing instead of pulling a lever. Both hypotheses were assessed separately by means of two default Bayes factors for paired, one-sided *t*-tests as outlined in Rouder et al. (2009) and Wetzels et al. (2009). Specifically, for the distribution for effect size under the alternative hypothesis we used a folded Cauchy(0,1) distribution. Exploratory analyses will vary the shape of this prior to probe the robustness of our conclusions.

Bayes factors quantify the support that the data provide for the null hypothesis vis-a-vis the alternative hypothesis. Support in favor of the alternative hypotheses constitutes support in favor of the effects reported by CB in their Experiment 2.

### Results

#### Bayes factor hypothesis tests

**Figure 7:**
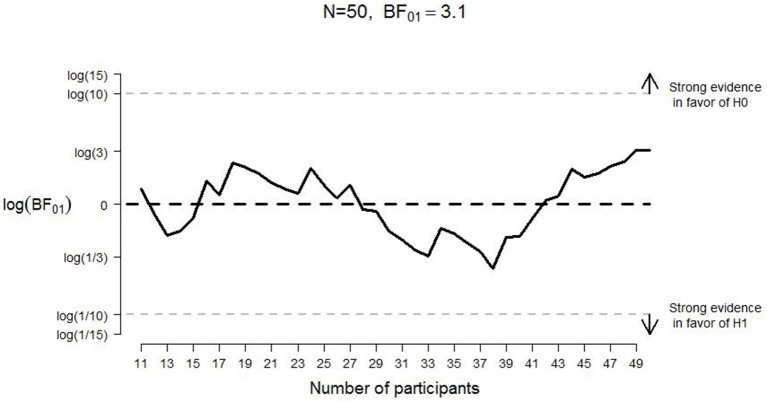
**Development of the log Bayes factor as a function of the number of participants for the positive words in Experiment 2**. *N* = sample size; *BF*_01_ = Bayes factor in favor of the null hypothesis; H0 = null hypothesis; H1 = alternative hypothesis. The horizontal black dashed line indicates complete ambiguous evidence, and the horizontal dashed gray lines indicate strong evidence either in favor of the null hypothesis [i.e., log(*BF*_01_) ≥ log(10)] or in favor of the alternative hypothesis [i.e., log(*BF*_01_) ≤ log(1/10)].

**Figure 8:**
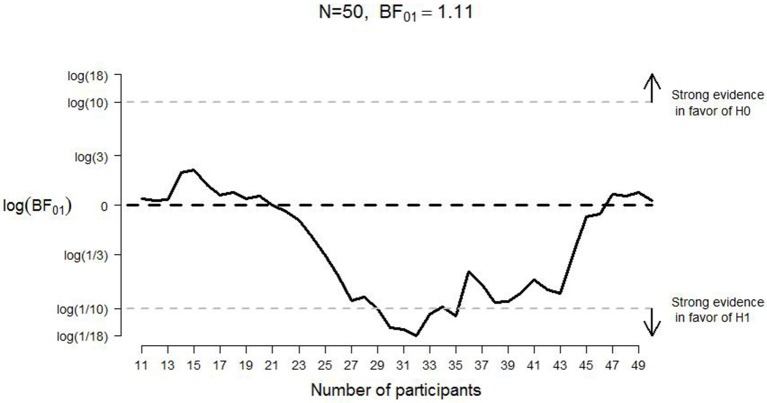
**Development of the log Bayes factor as a function of the number of participants for the negative words in Experiment 2**. *N* = sample size; *BF*_01_ = Bayes factor in favor of the null hypothesis; H0 = null hypothesis; H1 = alternative hypothesis. The horizontal black dashed line indicates complete ambiguous evidence, and the horizontal dashed gray lines indicate strong evidence either in favor of the null hypothesis [i.e., log(*BF*_01_) ≥ log(10)] or in favor of the alternative hypothesis [i.e., log(*BF*_01_) ≤ log(1/10)].

As Table 1 shows, for positive words participants were somewhat faster to pull (*M* = 562, *SE* = 13) the lever than to push it (*M* = 571, *SE* = 14). For negative words participants were somewhat faster to push the lever (*M* = 562, *SE* = 13) than to pull it (*M* = 574, *SE* = 12). For positive words, the comparison of the two lever movements yielded *BF*_*01*_ = 3.10; for negative words, it yielded *BF*_*01*_ = 1.11. In other words, for both positive and negative words we obtained “anecdotal” evidence (Jeffreys, 1961) in favor of the null hypothesis: the data are only about twice as likely under the null hypothesis as under the alternative hypothesis. Figures 7, 8 display the development of the log Bayes factor as a function of the number of participants for the positive words and the negative words, respectively. For positive words, the Bayes factor fluctuated heavily throughout the experiment, sometimes indicating anecdotal evidence in favor of the null hypothesis, and sometimes indicating anecdotal to moderate evidence for the alternative hypothesis. At the end of the data collection, the Bayes factor indicated moderate evidence for the absence of the congruency effect. For negative words, the Bayes factor initially indicated moderate to strong evidence in favor of the alternative hypothesis. However, after testing 33 participants, the Bayes factor support in favor of the alternative hypothesis started to lessen; at the end of data collection, the Bayes factor indicated that the evidence is almost perfectly ambiguous.

#### Exploratory analysis

**Figure 9:**
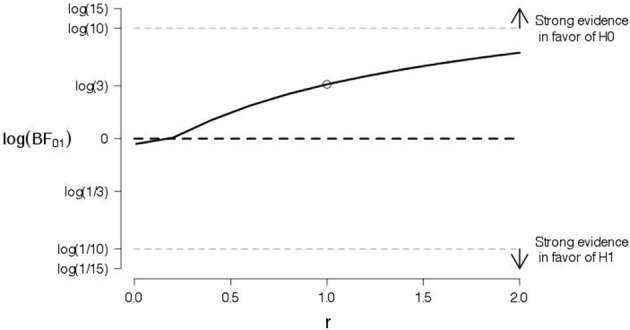
**A robustness analysis for the data of positive words from Experiment 2**. The log Bayes factor log(*BF*_01_) is plotted as a function of the scale parameter r of the Cauchy prior for the effect size under the alternative hypothesis. The dot indicates the result from the default prior, the horizontal black dashed line indicates complete ambiguous evidence, and the horizontal dashed gray lines indicate strong evidence either in favor of the null hypothesis [i.e., log(*BF*_01_) ≥ log(10)] or in favor of the alternative hypothesis [i.e., log(*BF*_01_) ≤ log(1/10)].

**Figure 10:**
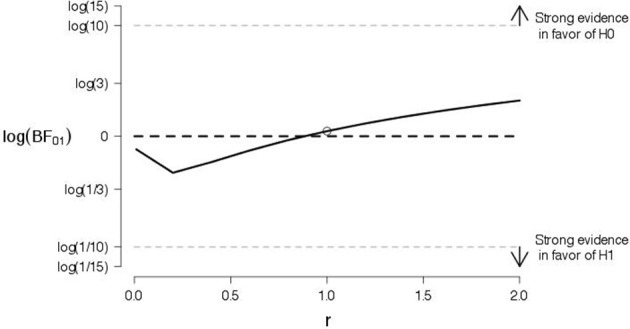
**A robustness analysis for the data of negative words from Experiment 2**. The log Bayes factor log(*BF*_01_) is plotted as a function of the scale parameter r of the Cauchy prior for the effect size under the alternative hypothesis. The dot indicates the result from the default prior, the horizontal black dashed line indicates complete ambiguous evidence, and the horizontal dashed gray lines indicate strong evidence either in favor of the null hypothesis [i.e., log(*BF*_01_) ≥ log(10)] or in favor of the alternative hypothesis [i.e., log(*BF*_01_) ≤ log(1/10)].

To probe the robustness of our conclusions, we varied the shape of the prior for the effect size under the alternative hypothesis. Figures 9, 10 show the log Bayes factor as a function of the scale parameter r of the Cauchy prior for the positive and negative words, respectively. The dot indicates the result from the default prior used in the preregistered data analysis. It is evident that, as the scale parameter r increases (i.e., the prior becomes progressively wider), the Bayes factor increasingly favors the null hypothesis. In addition, it is evident that, even under the prior setting that favors the alternative hypothesis most (i.e., scale parameter r very close to zero), the log Bayes factor is close to zero indicating ambiguous evidence.

For completeness, we also analyzed the data using a frequentist ANOVA (Table 1). Congruent lever movements were somewhat faster (*M* = 562 ms, *SE* = 13) than incongruent lever movements [*M* = 573 ms, *SE* = 13; *t*_(49)_ = 1.713, *p* = 0.093]. In line with this observation, a repeated measures 2 (Affective valence: Positive vs. Negative) × 2 (Lever movement: Pull vs. Push) ANOVA indicated a marginally significant Two-Way interaction [*F*_(1, 49)_ = 2.93, *p* = 0.09, η^2^_*p*_ = 0.057]: Pulling the lever with positive words was a little faster [*t*_(49)_ = 1.054, *p* = 0.297.] than pushing, whereas pulling the lever with negative words [*t*_(49)_ = 1.742, *p* = 0.088] was slower than pushing. This result perhaps provides a weak indication of congruency (i.e., the alternative hypothesis) as obtained in CB's Experiment 2. No other effects reached the 0.10 level of marginal significance.

The importance of the Two-Way interaction was also assessed with the help of a Bayesian ANOVA (Rouder et al., 2012) with participants as a random factor, which equals a repeated measures ANOVA. The Bayes factor of interest contrasts the full model that includes both the main effects and the interaction to a simpler model that includes only the main effects. The Bayes factor slightly favored the model without the interaction term (i.e., *BF*_*01*_ = 4.28), that is, the observed data are 4.28 times more likely under the model without the interaction compared to the model with the interaction.

**Figure 11:**
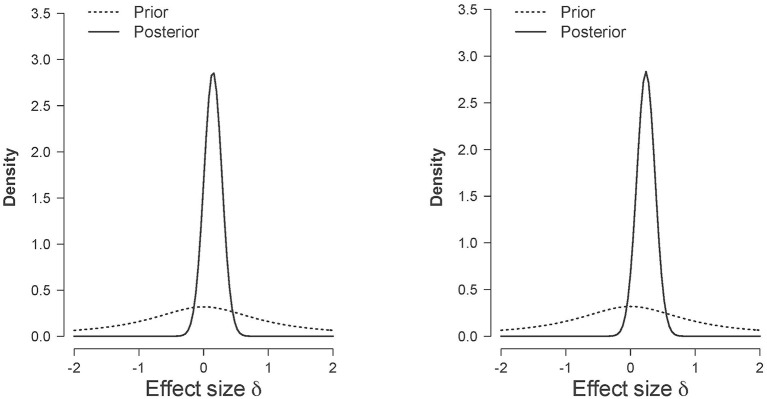
**Prior (dashed line) and posterior (solid line) distribution of the effect size δ in Experiment 2 for positive words (left panel) and negative words (right panel)**.

In sum, as was the case for Experiment 1, our preregistered Bayesian hypothesis tests yielded evidence in favor of the null hypothesis, although the strength of this evidence was rather modest. Figure 11 shows the posterior distribution of the effect size for the two preregistered comparisons (i.e., for positive words in the left panel, and for negative words in the right panel). It is evident, that in the case of both hypotheses, the posterior distribution is only slightly shifted to positive values. From a Bayesian perspective, the data certainly did not support the CB hypotheses. Our exploratory frequentist data analysis indicated some weak evidence in line with the original CB findings. This finding, however, is in contrast with the results obtained in the meta-analyses reported in Phaf et al. (2014, see also Laham et al., 2014) that seems to suggest the absence of any automatic congruency effect.

## Discussion and concluding comments

Our attempts to replicate the CB experiments did not succeed: for both replication attempts, the preregistered Bayesian hypothesis tests showed that the data provided more evidence for the null hypotheses than for the alternative hypotheses. The strength of this evidence is certainly not compelling, but the results do suggest that additional direct preregistered replications of the CB experiments are called for.

Using a frequentist ANOVA, exploratory analyses of Experiment 1 and Experiment 2 revealed a weak indication for congruency, expressed through interaction effects that were both marginally significant. These results were not, however, corroborated by a Bayesian ANOVA, which again provided weak evidence in favor of the absence of an interaction. This inconsistency arises as a result of the statistical peculiarities of *p*-values. As explained earlier, *p*-value-based inference overstates the amount of evidence against the null hypothesis because it fails to consider the extremeness of the data under the alternative hypothesis. Nevertheless, the evidence for the presence of the effects is weak to non-existent, even if we focus on the most favorable analysis (i.e., the exploratory frequentist ANOVA producing marginally significant results.

Although we attempted to duplicate the original experimental setup as accurately as possible there were of course small differences in the experimental setup and procedure that can maybe account for the differences in results. First, in our experimental setup participants had to move their hand a bit more than in the original setup. This could have resulted in less easy movements, for instance, maybe interfering with the congruency effect. When trying out both trajectories ourselves though we could not feel any more interference in the longer one we used than in the shorter one used by CB; hence, we do not believe this difference can explain the discrepant results. Moreover, latencies obtained in our experiment were faster than the original latencies suggesting that when any of such interference took place it certainly did not slow down our participants. Second, we used fewer words in our experiments (resp., 77 and 78 out of 92) than CB (i.e., 82 out of 92) assuming that targets used for practice trials in the original experiment were not used in the actual experiments and reported results. Of course this difference was due to our efforts to get our stimulus set to resemble the original stimulus set used by CB as close as possible so we do not think this difference can explain the discrepant results either. Third, in our experiments targets were randomly presented (Experiment 1) as well as semi-randomly (Experiment 2) whereas the original authors used a single randomly ordered list of words in both experiments. If this difference could explain the discrepant results we should probably conclude that the original findings are due to experimental noise alone but this would contrast again with our findings and the findings in Phaf et al. (2014, see also Laham et al., 2014). In sum, we do not think that these differences in experimental setup and procedure can account for the differences in results obtained by us and CB. But we are aware that we of course do not know all differences since we could rely only on specifics provided in the original report.

It seems clear that although we failed to replicate CB using our pre-registered Bayesian analyses we cannot conclude that there is no link between affective evaluation and approach-avoidance behavior. First, the evidence in favor of the null hypothesis is not compelling and stems from only two experiments. Second, using exploratory frequentist statistics we found weak evidence for this link. Third, a recent meta-analysis (Phaf et al., 2014; see also Laham et al., 2014) shows ample evidence for the presence of this link. Phaf et al. included studies on approach and avoidance behavior using different experimental paradigms (e.g., joystick, manikin) and, after correcting for publication bias, reported evidence for the presence of a moderate congruency effect between explicit affective evaluation and approach and avoidance behavior. Additionally, no evidence was obtained in this meta-analysis for such a direct link in case of implicit affective evaluation as in our Experiment 2.

In addition, we were also unable to replicate the main effect of evaluative judgment (Experiment 1) and affective meaning (Experiment 2) that was obtained by CB. In Experiment 1 of CB, negative evaluations were faster than positive evaluations; in Experiment 2 of CB, participants responded faster to negative than to positive words. In contrast, in our Experiment 1 we found that positive evaluations were faster than negative evaluations; in our Experiment 2, there was no evidence that affective meaning influenced lever movement in the absence of explicit affective evaluation. This finding and the aforementioned findings suggests that the pattern of results obtained by CB may be more fragile than previously thought.

### Conflict of interest statement

The authors declare that the research was conducted in the absence of any commercial or financial relationships that could be construed as a potential conflict of interest.

## Footnotes

^1^https://osf.io/wxigz/.

^2^We contacted John A. Bargh for help in designing our experiments.
