# Supplementary material for: On the automatic link between affect and tendencies to approach and avoid: Chen and Bargh (1999) revisited
Source: Front Psychol. 2015 Apr 2;6:335. doi: 10.3389/fpsyg.2015.00335 (PMC4382967; doi:10.3389/fpsyg.2015.00335)
Supplement: Supplementary file 1 [file Supplementarymaterials.PDF]

**Supplementary Material**

Paper: On the automatic link between affect and tendencies to approach and avoid: Chen and Bargh (1999) revisited. Rotteveel, M., Gierholz, A., Koch, G., van Aalst, C., Pinto, Y., Matzke, D., Steingroever, H., Verhagen, J., Beek, T.F., Selker, R., Sasiadek, A., Wagenmakers, E.J. (2015). *Frontiers in Psychology*, 6:335. Doi: 10.3389/fpsyg.2015.00335

Table 1. Affective evaluations of the Dutch targets used in Experiments 1 & 2 and of the corresponding original attitude objects from the Bargh et al. (1992) study as used in Chen and Bargh (1999).

| Original Attitude Object | Original Evaluation[1] | Translated Attitude Object (Dutch) | Replicated Evaluation[2] | Difference in Evaluation[3] |
|---|---|---|---|---|
| Alcoholism | -4.1 | Alcoholisme | -3.4 | -0.7 |
| Anchovies | -1.8 | Ansjovis | -0.4 | -1.4 |
| Aquarium | 1.9 | Aquarium | 1.5 | 0.4 |
| Baby | 3.5 | Baby | 2.5 | 1 |
| Basketball | 1.2 | Basketbal | 1.3 | -0.1 |
| Birthday | 3 | Verjaardag | 3.1 | -0.1 |
| Bombs | -4 | Bommen | -3.8 | -0.2 |
| Butterfly | 2.5 | Vlinder | 2.7 | -0.2 |
| Cake | 2.4 | Cake | 2.7 | -0.3 |
| Cancer | -4.4 | Kanker | -4.4 | 0 |
| Cavities | -3.2 | Gaatjes | -2.5 | -0.7 |
| Chocolate | 2.8 | Chocola | 3.3 | -0.5 |
| Circus | 1.9 | Circus | 0.7 | 1.2 |
| Clothes | 3.2 | Kleding | 3 | 0.2 |
| Cockroach | -3.8 | Kakkerlak | -3.1 | -0.7 |
| Coffee | 0.3 | Koffie | 1.7 | -1.5 |
| Crime | -3.9 | Misdaad | -3.4 | -0.5 |
| Dancing | 3.1 | Dansen | 3.5 | -0.4 |
| Death | -3.1 | Dood | -3.5 | 0.4 |
| Dentist | -1.1 | Tandarts | -0.3 | -0.8 |
| Disease | -3.9 | Ziekte | -3.6 | -0.3 |
| Divorce | -2.6 | Scheiding | -3.0 | 0.4 |
| Eagle | 1.9 | Adelaar | 1.3 | 0.6 |
| Exams | -1.2 | Examens | -0.9 | -0.3 |
| Flowers | 3.5 | Bloemen | 3.1 | 0.4 |
| Food | 3 | Voedsel | 3.4 | -0.4 |
| Football | 0.9 | Voetbal | 0.8 | 0.1 |
| Friday | 3.8 | Vrijdag | 3.1 | 0.7 |
| Friend | 4.4 | Vriend | 4.0 | 0.4 |
| Funeral | -3.6 | Begrafenis | -3.4 | -0.2 |
| Garbage | -3 | Vuilnis | -2.3 | -0.7 |
| Germs | -3.2 | Ziektekiemen | -3.1 | -0.1 |

| | | | | |
|---|---|---|---|---|
| Gift | 3.4 | Cadeau | 3.6 | -0.2 |
| Gold | 2.6 | Goud | 2.6 | - |
| Grease | -2.2 | Vet | - 2.1 | -0.1 |
| Guns | -2.2 | Geweren | -3.3 | 1.1 |
| Hangover | -2.9 | Misselijkheid | -2.6 | -0.3 |
| Hatred | -3.6 | Haat | -3.6 | - |
| Hawaii | 3.4 | Hawaii | 3.2 | 0.2 |
| Hell | -3.5 | Hel | -3.5 | - |
| Hitler | -4.2 | Hitler | -4.3 | 0.1 |
| Holiday | 3.6 | Vakantie | 4.4 | -0.8 |
| Hornet | -2.4 | Horzel | -2.4 | - |
| Ice cream | 3.2 | IJsje | 3.2 | - |
| Kitten | 2.8 | Kitten | 2.5 | 0.3 |
| Knives | -0.9 | Messen | -1.4 | 0.5 |
| Landlords | -0.6 | Huurbazen | -1.4 | 0.8 |
| Liver | -1.4 | Lever | -0.5 | -0.9 |
| Magazine | 2.7 | Tijdschrift | 2.1 | 0.6 |
| Monday | -0.8 | Maandag | -0.4 | -0.4 |
| Money | 2.8 | Geld | 2.7 | 0.1 |
| Mosquito | -2.9 | Mug | -2.9 | - |
| Movies | 3.2 | Films | 3.2 | - |
| Music | 4.2 | Muziek | 4.0 | 0.2 |
| Parade | 1.7 | Parade | 1.5 | 0.2 |
| Party | 3.2 | Feest | 3.8 | -0.6 |
| Pie | 1.7 | Taart | 3 | -1.3 |
| Pizza | 2.8 | Pizza | 3 | -0.2 |
| Radiation | -3.2 | Radiatie | -2.1 | -1.1 |
| Rattlesnake | -2.7 | Ratelslang | -2.3 | -0.4 |
| Reagan | -0.3 | Rutte | -0.4 | 0.1 |
| Recession | -1.7 | Recessie | -2.6 | 0.9 |
| Silk | 2.4 | Zijde | 1.9 | 0.5 |
| Smoking | -3.1 | Roken | -2.0 | -1.1 |
| Spider | -1.5 | Spin | -1.7 | 0.2 |
| Spinach | 0.7 | Spinazie | 2.1 | -1.4 |
| Sports | 2.3 | Sport | 2.7 | -0.4 |
| Strawberries | 2.9 | Aardbeien | 3.5 | -0.6 |
| Summer | 3.5 | Zomer | 4.4 | -0.9 |
| Sunshine | 3.9 | Zonneschijn | 4.4 | -0.5 |
| Swimming | 2.6 | Zwemmen | 2.8 | -0.2 |
| Taxes | -2.4 | Belasting | -1.6 | -0.8 |
| Television | 1.3 | Televisie | 1.7 | -0.4 |
| Toothache | -3.4 | Kiespijn | -3.3 | -0.1 |
| Virus | -3.5 | Virus | -3.5 | - |
| War | -4.1 | Oorlog | -4.2 | 0.1 |
| Weeds | -2 | Onkruid | -1.6 | -0.4 |
| Worms[4] | -1.9 | Wormen | -1.7 | -0.2 |

[1] Mean ratings on a scale from -5 (extremely negative) to 5 (extremely positive), taken from the Bargh et al. (1992) norming study. [2] Mean rating by a sample of the University of Amsterdam ($n$ = 130) on a translated version of the Bargh et al. (1992) scale from -5 (heel erg slecht) to 5 (heel erg goed). [3] Difference in mean ratings, calculated by subtracting the rates of our replication from the means of the original norming study (Bargh et al. 1992). Note that "Hangover" and "Reagan" were replaced. "Hangover" can be translated as " kater" but this is ambiguous in Dutch so we replaced it with "misselijkheid" which means "nausea". "Reagan" was replaced with "Rutte" who is the Dutch current prime minister. [4] This word was selected for Experiment 1 but was left out of the experiment due to a mistake.

Table 2. Affective evaluations of the Dutch targets that were not used in Experiments 1 & 2 and of the corresponding original attitude objects from the Bargh et al. (1992) study as used in Chen and Bargh (1999).

| Original Attitude Object | Original Evaluation[1] | Translated Attitude Object (Dutch) | Replicated Evaluation[2] | Difference in Evaluation[3] |
|---|---|---|---|---|
| Beer* | -0.5 | Bier | 1.3 | -1.8 |
| Clown* | 1.7 | Clown | -0.3 | 2 |
| Disco* | 0.6 | Disco | 2.4 | -1.8 |
| Dormitory | 1.1 | Slaapzaal | 0.0 | 1.1 |
| Fraternity* | 0 | Broederschap | 3 | -3 |
| Litter* | -3.1 | Rommel | -1.4 | -1.7 |
| Priest* | 0.9 | Priester | -1.2 | 2.1 |
| Rats* | -3.7 | Ratten | -2.1 | -1.6 |
| Russia | -1 | Rusland | 0.3 | -1.3 |
| Snow* | 2.5 | Sneeuw | 1 | 1.5 |
| Stereo* | 3.7 | Stereo | 2.1 | 1.6 |
| Storms* | 0.3 | Stormen | -1.2 | 1.5 |
| Tequila | -0.5 | Tequila | 0.2 | -0.7 |
| Vodka | -0.6 | Wodka | 0.3 | -0.9 |

[1] Mean ratings on a scale from -5 (extremely negative) to 5 (extremely positive), taken from the Bargh et al. (1992) norming study. [2] Mean rating by a sample of the University of Amsterdam ($n$ = 130) on a translated version of the Bargh et al. (1992) scale from -5 (heel erg slecht) to 5 (heel erg goed). [3] Difference in mean ratings, calculated by subtracting the rates of our replication from the means of the original norming study (Bargh et al. 1992). Attitude Objects have been excluded if either the difference exceeded 1.5 or the valence shifted from a negative to positive or from positive to negative. Target words marked with '*' were used in the practice trials.

# Appendix A

*Dutch instructions before practice trials in Experiment 1:*

Introduction

“We vragen je om de woorden die we zo dadelijk op het scherm laten zien zo snel mogelijk te categoriseren als “goed” of “slecht”. Gebruik de hendel die naast je staat om je antwoord mee te geven direct nadat het woord op het scherm is verschenen.”

Only in the congruent condition:

“Als het woord “goed” is vragen we je de hendel naar je toe te bewegen en als het woord “slecht” is vragen we je de hendel van je af te bewegen. Laat nadat je je antwoord hebt gegeven de hendel los zodat die weer naar het middelpunt kan terugkeren. Je gaat nu eerst een paar keer oefenen voordat het experiment begint. Je zult tijdens deze oefentrials steeds kunnen zien of je het woord als “goed” of “slecht” hebt beoordeeld. Deze feedback zal tijdens het experiment echter afwezig zijn. Mocht je na de oefentrials nog vragen hebben stel deze dan gerust aan de proefleider. Veel succes!”

Only in the incongruent condition:

“Als het woord “goed” is vragen we je de hendel van je af te bewegen en als het woord “slecht” is vragen we je de hendel naar je toe te bewegen. Laat nadat je je antwoord hebt gegeven de hendel los zodat die weer naar het middelpunt kan terugkeren. Je gaat nu eerst een paar keer oefenen voordat het experiment begint. Je zult tijdens deze oefentrials steeds kunnen zien of je het woord als “goed” of “slecht” hebt beoordeeld. Deze feedback zal tijdens het experiment echter afwezig zijn. Mocht je na de oefentrials nog vragen hebben stel deze dan gerust aan de proefleider. Veel succes!”

*Translated instructions from Dutch before practice trials of Experiment 1:*

Introduction

“We will show you some words on the computer screen and ask you to categorize these words as fast as possible as being “good” or “bad”. Use the lever beside you to give your response immediately after the word appears.”

Only in the congruent condition:

“If the word is “good” please pull the lever toward yourself and when the word is “bad” push the lever away instead. Please release the lever after responding in order to let it return to mid position. Before we start with the experiment you get a few practice trials. During these practice trials you will see whether you classified the word as being “good” or “bad”. Note that there will be no feedback like this during the actual experiment. If you have any questions left after practice please feel free to ask the experimenter. Good luck!”

Only in the incongruent condition:

“If the word is “good” please push the lever away and when the word is “bad” pull the lever back toward instead. Please release the lever after responding in order to let it return to mid position. Before we start with the experiment you get a few practice trials. During these practice trials you will see whether you classified the word as being “good” or “bad”. Note that there will be no feedback like this during the actual experiment. If you have any questions left after practice please feel free to ask the experimenter. Good luck!”

# Appendix B

*Dutch instructions before practice trials in Experiment 2:*

## Introduction a

“We vragen je om zo snel mogelijk te reageren zodra je een woord op het scherm ziet verschijnen. Dit kun je doen door de hendel die naast je staat zo snel mogelijk van je af te bewegen. Laat nadat je dit hebt gedaan de hendel los zodat die weer naar het middelpunt kan terugkeren. Je gaat nu eerst een paar keer oefenen. Als je daarna nog vragen hebt stel die dan gerust aan de proefleider. Veel succes!”

## Introduction b

“We vragen je om zo snel mogelijk te reageren zodra je een woord op het scherm ziet verschijnen. Dit kun je doen door de hendel die naast je staat zo snel mogelijk naar je toe te bewegen. Laat nadat je dit hebt gedaan de hendel los zodat die weer naar het middelpunt kan terugkeren. Je gaat nu eerst een paar keer oefenen. Als je daarna nog vragen hebt stel die dan gerust aan de proefleider. Veel succes!”

*Translated instructions from Dutch before practice trials in Experiment 2:*

Introduction a

“We ask you to respond as fast as possible after word appearance by pushing the lever away. Please release the lever after responding in order to let it return to mid position. First you get a few practice trials and if you have any questions left please feel free to ask the experimenter. Good luck!”

Introduction b

“We ask you to respond as fast as possible after word appearance by pulling the lever toward yourself. Please release the lever after responding in order to let it return to mid position. First you get a few practice trials and if you have any questions left please feel free to ask the experimenter. Good luck!”
